# Suppression of Natural Killer Cell Activity by Regulatory NKT10 Cells Aggravates Alcoholic Hepatosteatosis

**DOI:** 10.3389/fimmu.2017.01414

**Published:** 2017-10-30

**Authors:** Kele Cui, Guoxiu Yan, Xiaodong Zheng, Li Bai, Haiming Wei, Rui Sun, Zhigang Tian

**Affiliations:** ^1^The CAS Key Laboratory of Innate Immunity and Chronic Disease and Institute of Immunology, School of Life Sciences and Medical Center, University of Science & Technology of China, Hefei, China; ^2^Anhui Province Hospital Affiliated Anhui Medical University, Hefei, China; ^3^Collaborative Innovation Center for Diagnosis and Treatment of Infectious Diseases, State Key Laboratory for Diagnosis and Treatment of Infectious Diseases, First Affiliated Hospital, College of Medicine, Zhejiang University, Hangzhou, China

**Keywords:** alcoholic fatty liver, natural killer cells, invariant natural killer T cells, interferon-γ, interleukin-10, interaction

## Abstract

We and others have found that the functions of hepatic natural killer (NK) cells are inhibited but invariant NKT (iNKT) cells become activated after alcohol drinking, leaving a possibility that there exists interplay between NK cells and iNKT cells during alcoholic liver disease. Here, in a chronic plus single-binge ethanol consumption mouse model, we observed that NK cells and interferon-γ (IFN-γ) protected against ethanol-induced liver steatosis, as both wild-type (WT) mice treated with anti-asialo GM1 antibody and IFN-γ-deficient GKO mice developed more severe alcoholic fatty livers. As expected, IFN-γ could directly downregulate lipogenesis in primary hepatocytes *in vitro*. On the contrary, iNKT cell-deficient Jα18^−/−^ or interleukin-10 (IL-10)^−/−^ mice showed fewer alcoholic steatosis, along with the recovered number and IFN-γ release of hepatic NK cells, and exogenous IL-10 injection was sufficient to compensate for iNKT cell deficiency. Furthermore, NK cell depletion in Jα18^−/−^ or IL-10^−/−^ mice caused more severe hepatosteatosis, implying NK cells are the direct effector cells to inhibit liver steatosis. Importantly, adoptive transfer of iNKT cells purified from normal but not IL-10^−/−^ mice resulted in suppression of the number and functions of NK cells and aggravated alcoholic liver injury in Jα18^−/−^ mice, indicating that IL-10-producing iNKT (NKT10) cells are the regulators on NK cells. *Conclusion*: Ethanol exposure-triggered NKT10 cells antagonize the protective roles of NK cells in alcoholic hepatosteatosis.

## Introduction

Excessive alcohol consumption is a major etiologic factor in chronic liver disease worldwide. The mechanisms underlying the formation of alcoholic liver disease (ALD) are complex and polyfactorial and involve alcohol metabolite-induced hepatotoxicity, oxidative stress, and the overactivity or dysregulation of the innate immune system caused liver damage (1). The liver, an organ with predominant innate immunity, is enriched with innate immune cells, such as Kupffer cells, natural killer (NK) cells, NKT cells, and neutrophils, which have been implicated in ALD (2).

As a key component of innate immunity in the liver, NK cells play important roles in antiviral infection and tumor immunosurveillance through their natural cytotoxicity, such as degranulation to directly kill target cells or the secretion of large amounts of cytokines, such as interferon-γ (IFN-γ) and tumor necrosis factor-α, which act on other immune cells (3–6). NK cells reportedly protect against alcoholic liver fibrosis through the killing of activated hepatic stellate cells (HSCs) which express elevated NK cell-activating ligands, as well as by producing IFN-γ, which induces the cell cycle arrest and apoptosis of HSCs (7). However, alcohol is considered an immunosuppressive agent that inhibits NK cell activities, although the molecular mechanisms underlying the suppression of NK cell function are not yet clear (8, 9). In addition, little is known about the roles of NK cells in the early stages of ALD, such as alcoholic hepatosteatosis.

The liver is also enriched with NKT cells, a complicated, heterogeneous subset of specialized, self-reactive T cells with innate and adaptive immune properties (10, 11). Based on the expression of the invariant T cell receptor α-chain, NKT cells can be divided into two groups: conventional type I NKT cells [also called invariant NKT (iNKT) cells] and type II NKT cells (12). In acute liver injury, NKT cell activation induces hepatocyte damage, whereas in chronic hepatic disease, different NKT cell subsets may exert opposite functions as the result of their heterogeneity (13, 14). A recent study showed that pretreatment with α-GalCer could prompt a subset of iNKT cells to produce large amounts of interleukin-10 (IL-10) and to acquire regulatory cellular characteristics, subsequently impairing anti-tumor immune responses and alleviating the pathogenesis of experimental autoimmune encephalomyelitis. This group of iNKT cells with immunomodulatory properties is referred to as NKT10 cells, which represent a distinct iNKT cell subset (15). However, whether these NKT10 cells play a role in ALD remains unknown.

We and others previously reported that Kupffer cell-derived NLRP3 inflammasome activation and IL-1β maturation-induced hepatic iNKT cell accumulation are critical in the pathophysiologic process of alcoholic hepatosteatosis, likely involving neutrophil recruitment-mediated liver injury (16–18). In the present study, we demonstrated that NK cells play essential protective roles against liver steatosis through the IFN-γ-induced downregulation of fat metabolism-associated genes in hepatocytes in a chronic plus single-binge ethanol consumption mouse model, which can be inhibited by a IL-10-producing iNKT cell subset (also called NKT10 cells), suggesting an interplay between NK cells and NKT10 cells during ALD pathogenesis.

## Materials and Methods

### Mice

Female C57BL/6 mice (8–10 weeks old) were purchased from the Shanghai Laboratory Animal Center at the Chinese Academy of Sciences (Shanghai, China). The female Jα18^−/−^, IL-10^−/−^, and IFN-γ-deficient GKO mice on the C57BL/6 background were previously described (19–21). All mice were maintained under specific pathogen-free conditions with a 12-h light/dark cycle.

### Animal Model

A mouse model of chronic plus single-binge ethanol consumption (shortened to the chronic-binge model) was established as previously described (22). For each chronic-binge model experiment, the mice were gavaged late at night (23:30) or early in the morning (7:30) and were then euthanized at the indicated time points after gavage.

### Reagents

Recombinant murine IL-10 was purchased from PeproTech (Rocky Hill, NJ, USA).

### Flow Cytometry and Antibody Staining

The following fluorescence-labeled monoclonal antibodies against cell-surface antigens were used: fluorescein isothiocyanate-anti-CD107a, peridinin chlorophyll-Cy5.5-anti-NK1.1, and allophycocyanin-anti-CD3 were purchased from BD Biosciences (San Jose, CA, USA); phycoerythrin (PE)-CY7-anti-IFN-γ was purchased from eBioscience (San Diego, CA, USA); anti-PE microbeads were purchased from Miltenyi Biotec (Bergisch Gladbach, NRW, Germany); and the PE-anti-CD1d tetramer was obtained from the National Institutes of Health Tetramer Core Facility (Atlanta, GA, USA). The cells were stained with the indicated antibodies and analyzed using an FACSCalibur flow cytometer (BD Biosciences, NJ, USA) and FlowJo 7.6.1 software.

### NK Cell Depletion

For NK cell depletion, the mice were injected with 50 µg of anti-asialo GM1 (AsGM1) (rabbit) antibody (Wako Co., Tokyo, Japan) once every 3 days. Rabbit IgG (ZSGB-BIO, Peking, China) was used as a negative control.

### Cell Purification and Adoptive Transfer

Single-cell suspensions of liver mononuclear cells (MNCs) from 10-day ethanol diet-fed wild-type (WT) or IL-10^−/−^ mice were essentially prepared as previously described (23). iNKT cells were labeled with PE-anti-CD1d tetramer primary antibody and anti-PE microbeads and were isolated by magnetic-activated cell sorting. The purity of the sorted iNKT cell populations was >90%, verified by post-sort flow cytometric analysis. For adoptive cell transfer, Jα18^−/−^ mice undergoing the chronic-binge model were given intrasplenic injections of 5 × 10^5^ purified hepatic iNKT cells in 100 µL of saline immediately following gavage; the controls were injected with an equal volume saline.

### Stimulation of Primary Hepatocytes *In Vitro*

Primary hepatocytes were isolated using a two-step collagenase perfusion method as previously described (24). For hepatocyte stimulation, 2.5 × 10^6^ liver parenchymal cells were placed onto rat tail collagen-coated 12-well tissue-culture plates in 1 mL of Dulbecco’s modified Eagle medium (DMEM) complete medium supplemented with 10% fetal calf serum, 2 mM L-glutamine, 10 mM HEPES, 10 mM 2-mercaptoethanol, and 100 IU/mL penicillin/streptomycin and were subsequently cultured in a 5% CO_2_ atmosphere in a 37°C incubator for 5 h before replacing the medium with incomplete DMEM. After 4 h, the incomplete medium was removed, and the cells were cultured in fresh complete DMEM and stimulated with 0, 1, 10, or 100 ng/mL IFN-γ as indicated for 12 h. Subsequently, the cells were collected, and the mRNA levels of fat metabolism-associate genes were detected by quantitative PCR (q-PCR).

### RNA Isolation and q-PCR

Total mRNA from liver tissues, cultured hepatocytes, or purified iNKT cells was extracted and reverse-transcribed as previously described (16). The gene expression levels were measured by q-PCR using the commercially available SYBR Premix ExTaq (TaKaRa Biotechnology, Dalian, China) according to the manufacturer’s instructions. The following primer sequences were used: β-actin, sense 5′-TGGAATCCTGTGGCATCCATGAAA-3′ and antisense 5′-TAAAACGCAGCTCAGTAACAGTCCG-3′; IL-10, sense 5′-TGCTATGCTGCCTGCTCTTACTGA-3′ and antisense 5′-CCTGCTCCACTGCCTTGCTCTTAT-3′; SREBP-1, sense 5′-GAGGCCAAGCTTTGGACCTGG-3′ and antisense 5′-CCTGCCTTCAGGCTTCTCAGG-3′; FAS, sense 5′-ATTGCATCAAGCAAGTGCAG-3′ and antisense 5′-GAGCCGTCAAACAGGAAGAG-3′; ACC, sense 5′-TGAAGGGCTACCTCTAATG-3′ and antisense 5′-TCACAACCCAAGAACCAC-3′; SCD-1, sense 5′-CTGCCTCTTCGGGATTTTCTACT-3′ and antisense 5′-GCCCATTCGTACACGTGATTC-3′; FAT, sense 5′-AGATGCAGCCTCATTTCCAC-3′ and antisense 5′-GCCTTGGATGGAAGAACAAA-3′; GPAT, sense 5′-TTCCTCCTTCATCACAAAGAAGTCTT-3′ and antisense 5′-TGGCCTGTCTGCTCCTCTACA-3′. All gene-of-interest expression levels were calculated relative to the housekeeping gene β-actin.

### Statistical Analysis

Student’s *t*-test was performed to analyze the significance of differences between two groups when appropriate. Values of *P* < 0.05 were considered statistically significant.

## Results

### NK Cells and IFN-γ Play Protective Roles against Alcoholic Hepatosteatosis

**Figure 1:**
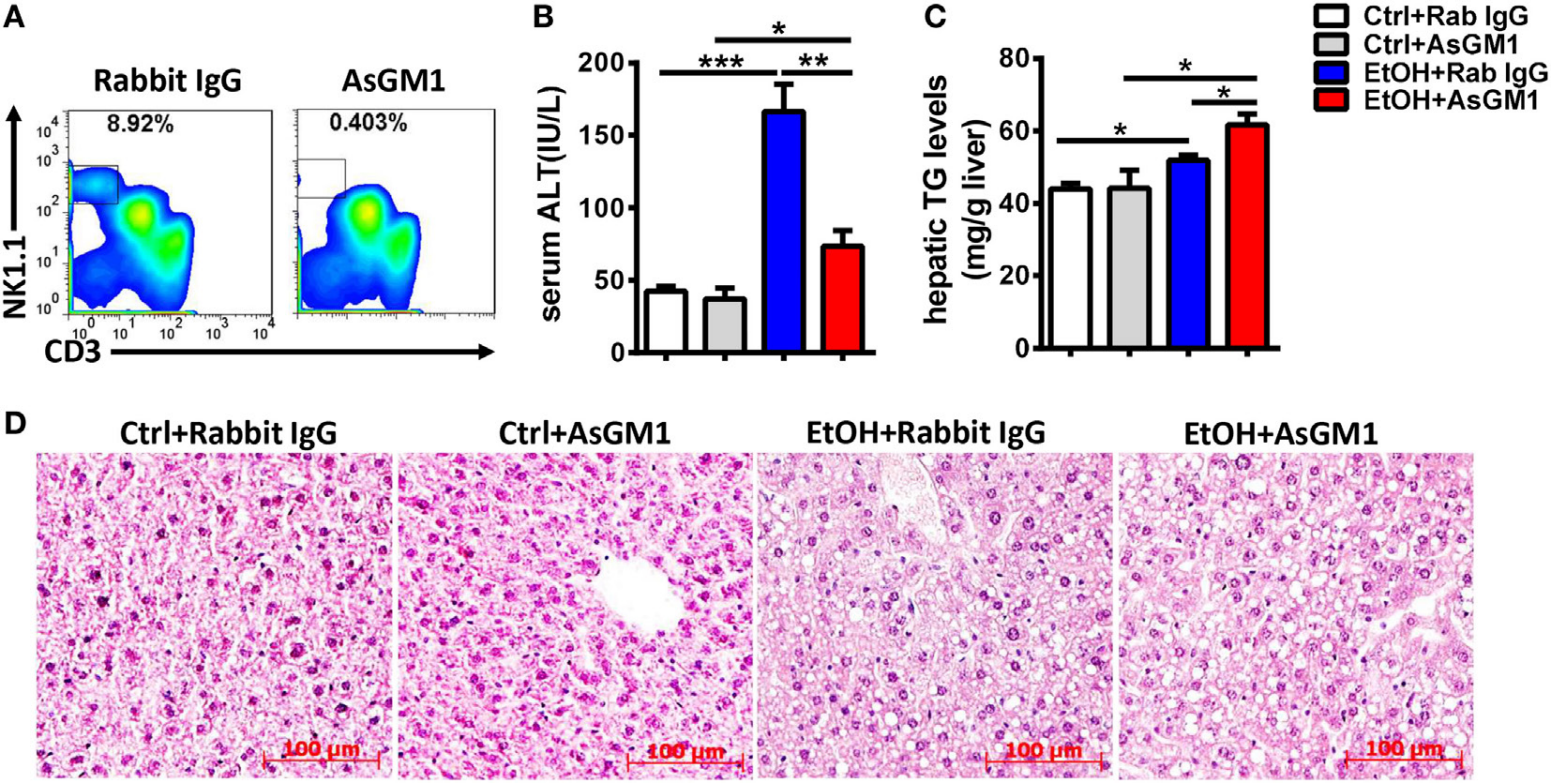
Deficiency of natural killer (NK) cells promoted liver steatosis but ameliorated inflammation in a chronic-binge model. Pair- or ethanol-fed wild-type C57BL/6 mice were treated with anti-asialo GM1 or control rabbit IgG once every 3 days as described in Section “Materials and Methods” to deplete NK cells. The mice were euthanized 9 h after gavage. **(A)** Liver mononuclear cells were isolated, and the depletion efficiency was verified by flow cytometry; liver injury and steatosis were evaluated based on **(B)** serum alanine aminotransferase and **(C)** liver triglyceride levels and **(D)** liver tissue hematoxylin and eosin staining (original magnification, 200×). The data are representative of two independent experiments and are shown as the mean ± SEM (*n* = 4–6). *P < 0.05; **P < 0.01; ***P < 0.005.

**Figure 2:**
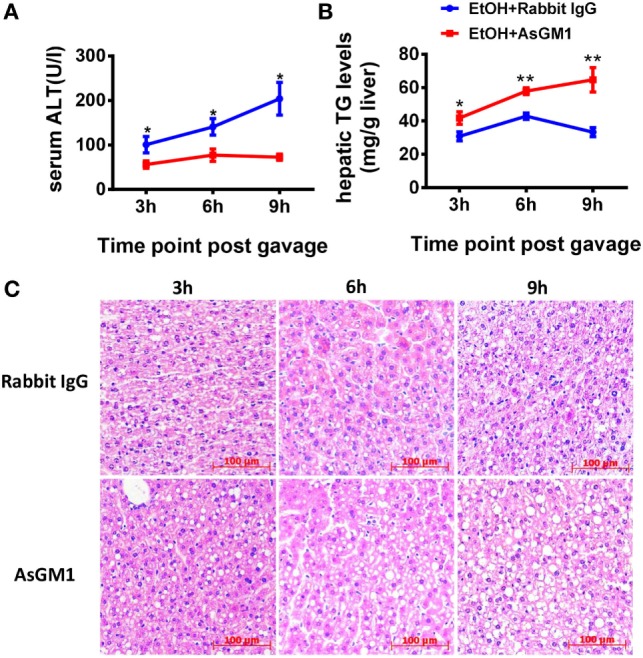
Deficiency of natural killer (NK) cells promoted liver steatosis but ameliorated inflammation at different time points. **(A)** Pair- or ethanol-fed wild-type C57BL/6 mice were treated with anti-asialo GM1 or control rabbit IgG once every 3 days as described in Section “Materials and Methods” to deplete NK cells. The mice were euthanized 3, 6, or 9 h after gavage. Liver injury and steatosis were evaluated based on **(A)** serum alanine aminotransferase and **(B)** liver triglyceride levels and **(C)** liver tissue hematoxylin and eosin staining (original magnification, 200×). The data are representative of two independent experiments and are shown as the mean ± SEM (*n* = 4–6). *P < 0.05; **P < 0.01.

To investigate whether NK cells play a role in the early stages of ALD, such as alcohol-induced hepatosteatosis, we depleted the NK cells in the C57BL/6 mice undergoing chronic-binge model using anti-AsGM1 antibody. Flow cytometric analysis of liver MNCs confirmed the efficient depletion of CD3^−^NK1.1^+^ NK cells (Figure 1A). Interestingly, compared with rabbit IgG-treated control mice, mice receiving anti-AsGM1 antibody displayed decreased serum alanine aminotransferase (ALT) levels but higher hepatic triglyceride (TG) levels, accompanied with pan-lobular steatosis stained with hematoxylin and eosin (H&E) 9 h after gavage (Figures 1B–D), indicating that NK cells play both a protective role against steatosis and a stimulatory role in hepatocyte injury. Furthermore, we observed that hepatic steatosis progressively increased and that liver damage was gradually alleviated in the AsGM1-treated mice at 3, 6, and 9 h after ethanol gavage compared with control mice, confirming that NK cells exert different functions in liver steatosis and injury (Figure 2).

**Figure 3:**
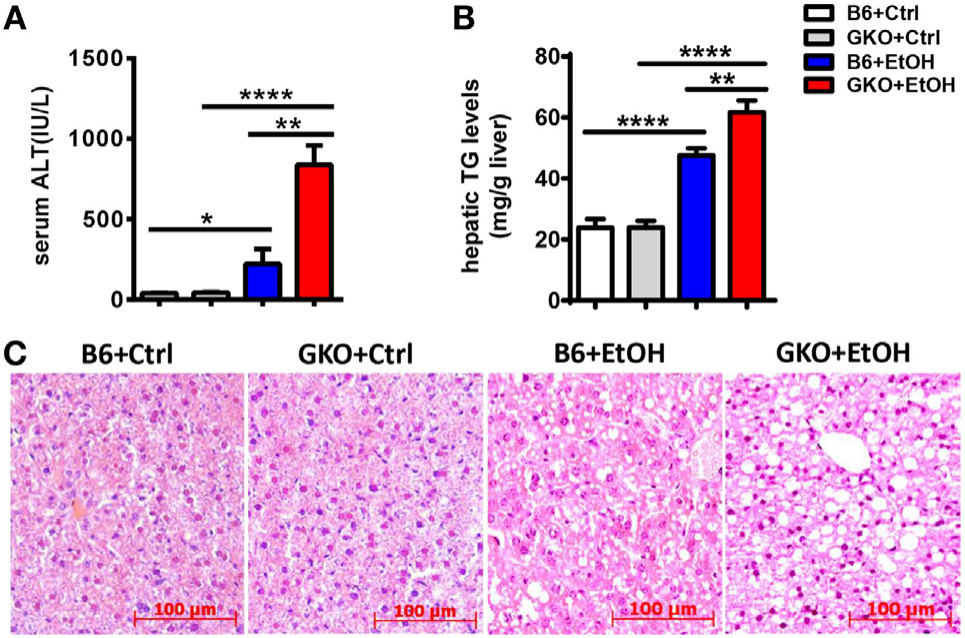
Deficiency of interferon-γ promoted ethanol-induced liver injury. **(A–C)** wild-type or GKO mice were fed control or ethanol diets for 10 days and received a gavage. The mice were sacrificed 9 h after gavage, and liver injury and steatosis were evaluated based on **(A)** serum alanine aminotransferase and **(B)** liver triglyceride levels and **(C)** liver tissue hematoxylin and eosin staining (original magnification, 200×). The data are representative of more than three independent experiments and are shown as the mean ± SEM (*n* = 4–11). *P < 0.05; **P < 0.01; ****P < 0.0001.

**Figure 4:**
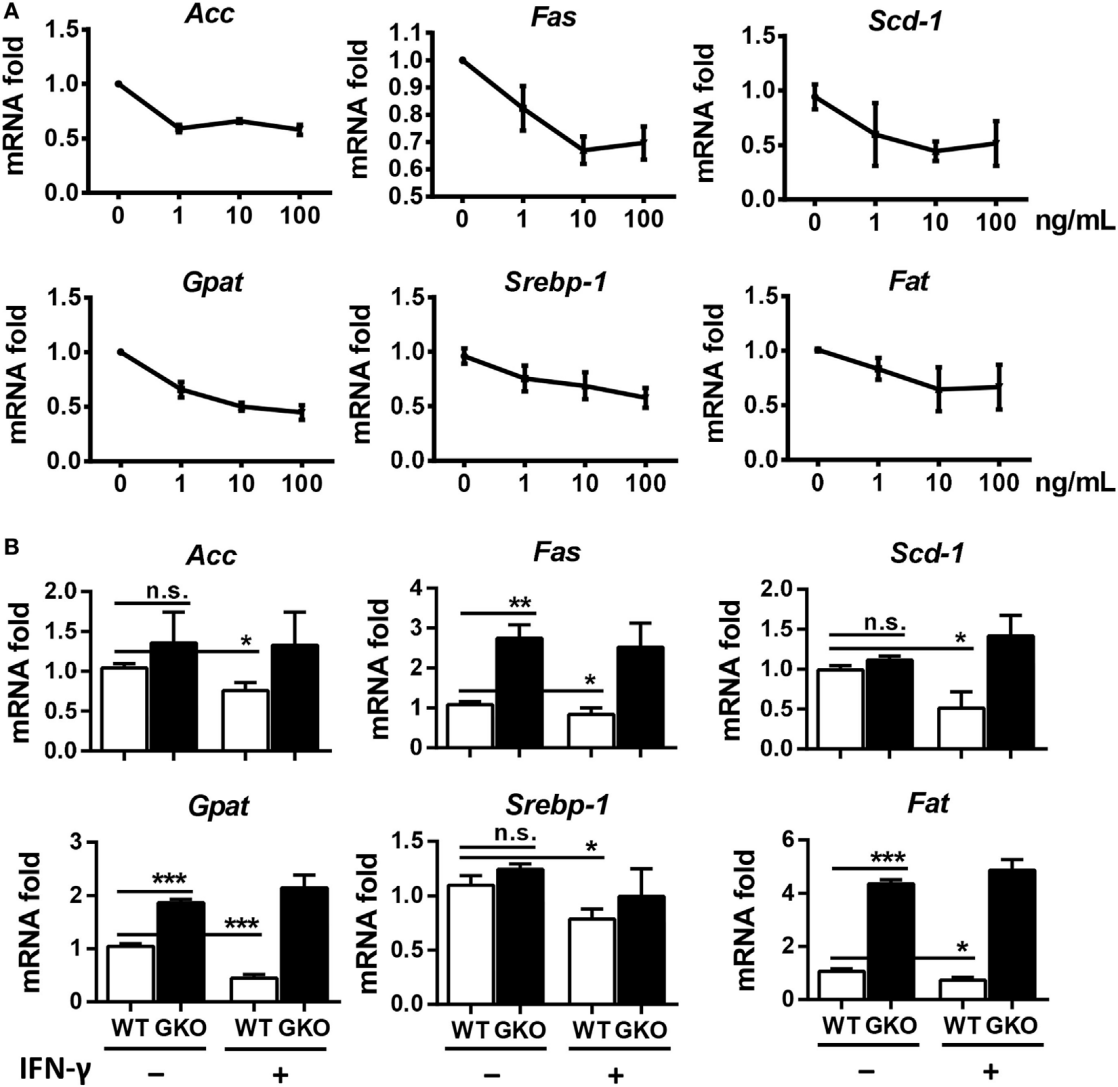
Interferon-γ (IFN-γ) inhibits the expression of fat synthesis-associated genes in hepatocytes. **(A)** Primary hepatocytes (2 × 10^5^) from the livers of wild-type (WT) mice were isolated and stimulated with 0, 1, 10, or 100 ng/mL IFN-γ, and the cells were collected to evaluate the mRNA levels of *ACC, FAS, SCD-1, GPAT, SREBP-1*, and *FAT* after 12 h culture. **(B)** Primary hepatocytes (2 × 10^5^) from the livers of WT mice and GKO mice were isolated and stimulated with or without 100 ng/mL IFN-γ, and the cells were collected to evaluate the mRNA levels of *ACC, FAS, SCD-1, GPAT, SREBP-1*, and *FAT* after 12 h culture. The data are representative of two independent experiments and are shown as the mean ± SEM (*n* = 3). *P < 0.05; **P < 0.01; ***P < 0.005.

Since IFN-γ is an important effector molecule produced by NK cells, we hypothesized that IFN-γ was also involved in alcohol-induced hepatosteatosis. To test this hypothesis, we generated a chronic-binge model in WT C57BL/6 and IFN-γ-deficient GKO mice. Notably, GKO mice displayed significantly more severe liver damage and steatosis than WT mice (Figures 3A–C), suggesting a critical role for IFN-γ in the pathophysiologic process of alcoholic hepatosteatosis. Imbalanced fat metabolism, such as decreased mitochondrial lipid oxidation and enhanced TG synthesis, results in fat accumulation in hepatocytes. To investigate whether IFN-γ directly influences fat metabolism, primary hepatocytes were purified and cultured in the presence or absence of IFN-γ *in vitro*. Indeed, INF-γ could clearly downregulate the expression levels of several lipogenesis (*Srebp-1, Fas, Acc, Gpat*, and *Scd1*) and fatty acid uptake (*Fat*) genes in hepatocytes in a dose-dependent manner (Figure 4A). In addition, the mRNA levels of *Fas, Gpat*, and especially *Fat* in the naïve hepatocytes from GKO mice were obviously higher than that from WT mice, and this high expression were not influenced by exogenous IFN-γ stimulation, indicating that the IFN-γ-deficient hepatocytes have stronger fatty acid uptake and fat synthesis abilities and will be more sensitive when exposed to ethanol (Figure 4B). Taken together, these data demonstrated that NK cells and IFN-γ exert protective functions against alcohol-induced fatty liver, likely through affecting the expression levels of lipogenesis-associated genes in hepatocytes.

### iNKT Cells Promote Alcoholic Hepatosteatosis by Inhibiting Liver Accumulation and IFN-γ Release of NK Cells

**Figure 5:**
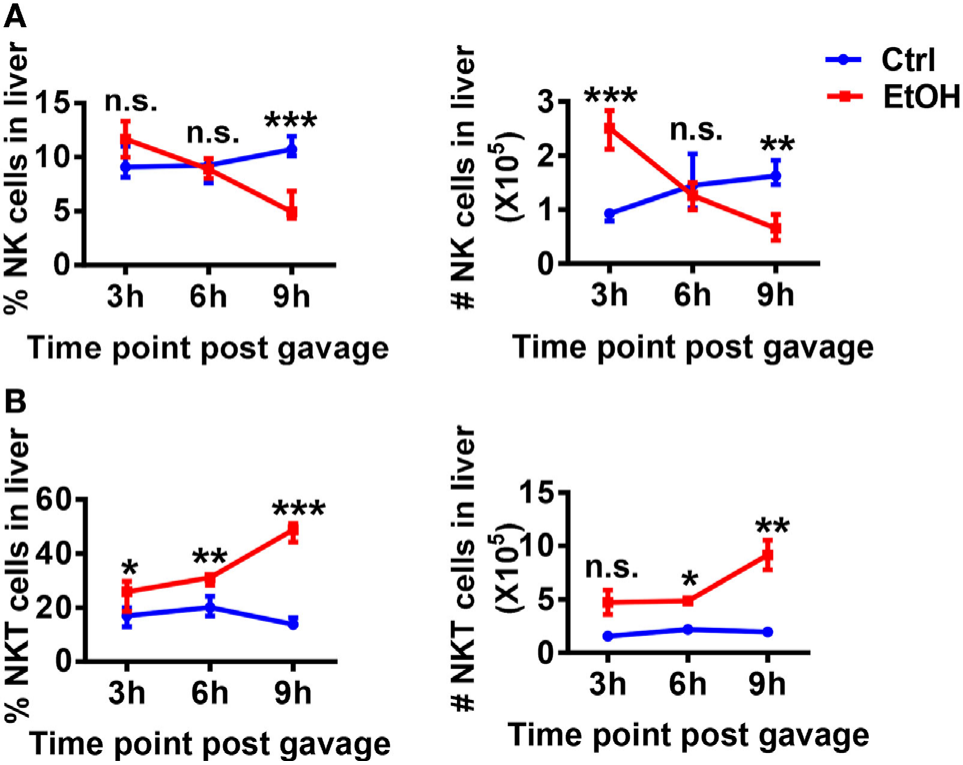
Hepatic natural killer (NK) and NKT cells display negatively correlated dynamics in the chronic-binge model. C57BL/6 mice were fed control or ethanol diets for 10 days plus one intragastric dose of EtOH (5 g/kg body weight) or isocaloric dextran-maltose, followed by euthanasia 3, 6, or 9 h after gavage. Liver mononuclear cells were isolated, and the percentages and absolute numbers of **(A)** CD3^−^NK1.1^+^NK cells and **(B)** CD3^+^NK.1^+^T cells among all leukocytes in liver were detected by flow cytometry and were statistically analyzed. The data are representative of more than three independent experiments and are shown as the mean ± SEM (*n* = 3–6). *P < 0.05; **P < 0.01; ***P < 0.005.

Natural killer cell function was reportedly suppressed rather than activated in ALD (8). The analysis of hepatic NK cell kinetics in WT mice in the chronic-binge model revealed that the frequency and absolute number of NK cells in isolated liver MNCs were progressively decreased at 3, 6, and 9 h after ethanol gavage compared with those in control mice (Figure 5A). Interestingly, the frequency and absolute number of NKT cells in hepatic MNCs were gradually increased, showing opposite kinetics compared with NK cells (Figure 5B). Because iNKT cells were previously shown to promote chronic-plus-binge alcoholic hepatosteatosis, we hypothesized that the accumulated and activated iNKT cells in the liver aggravated alcoholic fatty liver by inhibiting the protective functions of NK cells.

**Figure 6:**
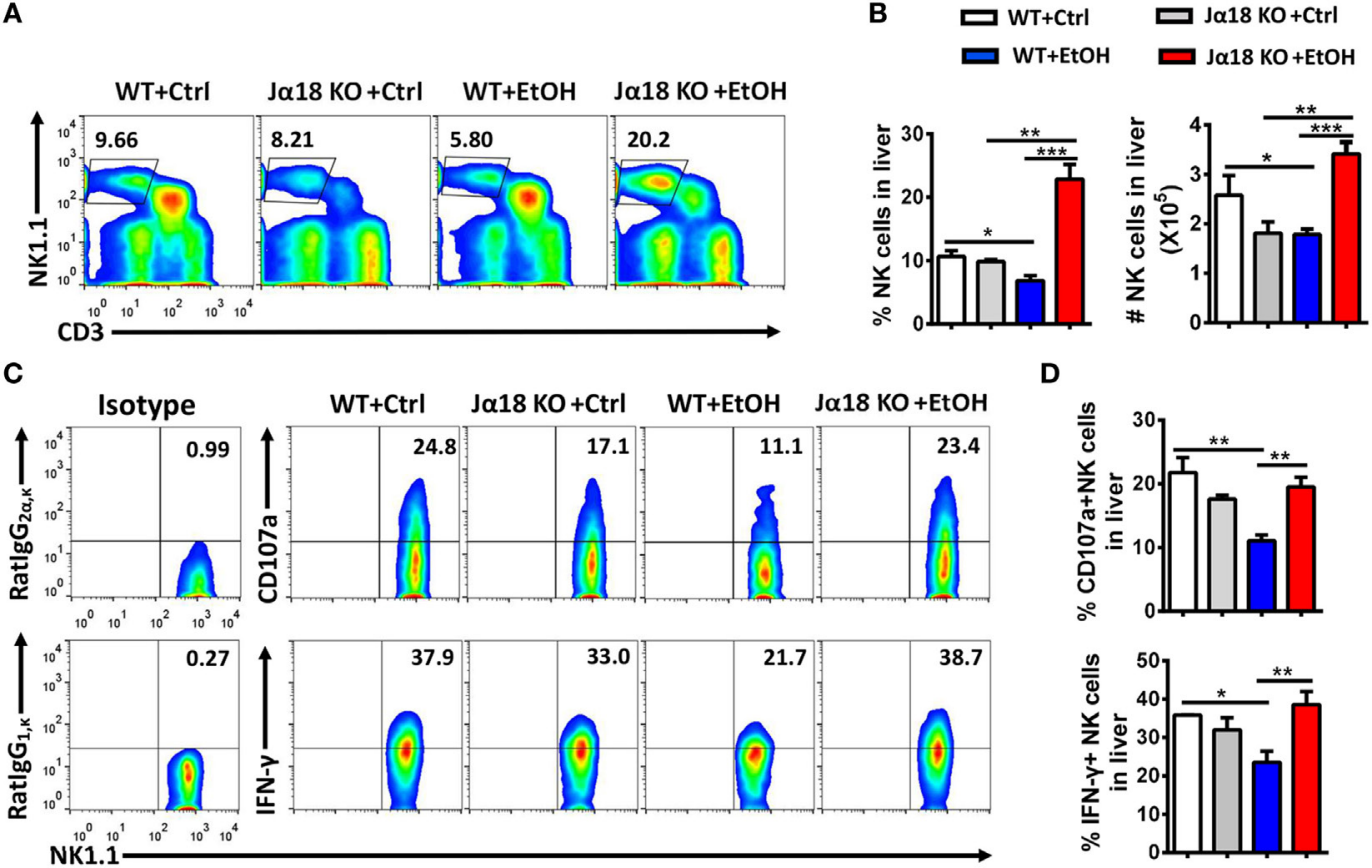
The number and function of natural killer (NK) cells were markedly increased in Jα18^−/−^ mice. **(A–D)** Wild-type or Jα18^−/−^ mice were fed control or ethanol diets for 10 days and subsequently received gavage. The mice were sacrificed 9 h after gavage, and the liver mononuclear cells were then isolated. **(A)** The frequency of CD3^−^NK1.1^+^ NK cells among all leukocytes in the liver was analyzed by flow cytometry. **(B)** Statistical analysis of the percentage and absolute number of the NK cells shown in **(A)**. **(C)** Representative CD107a expression (without PMA stimulation) and interferon-γ (IFN-γ) release (with PMA stimulation) in hepatic NK cells. **(D)** Statistical analysis of the percentages of CD107a^+^ and IFN-γ^+^ NK cells shown in **(C)**. The data are representative of more than three independent experiments and are shown as the mean ± SEM (*n* = 3–6). *P < 0.05; **P < 0.01; ***P < 0.005.

**Figure 7:**
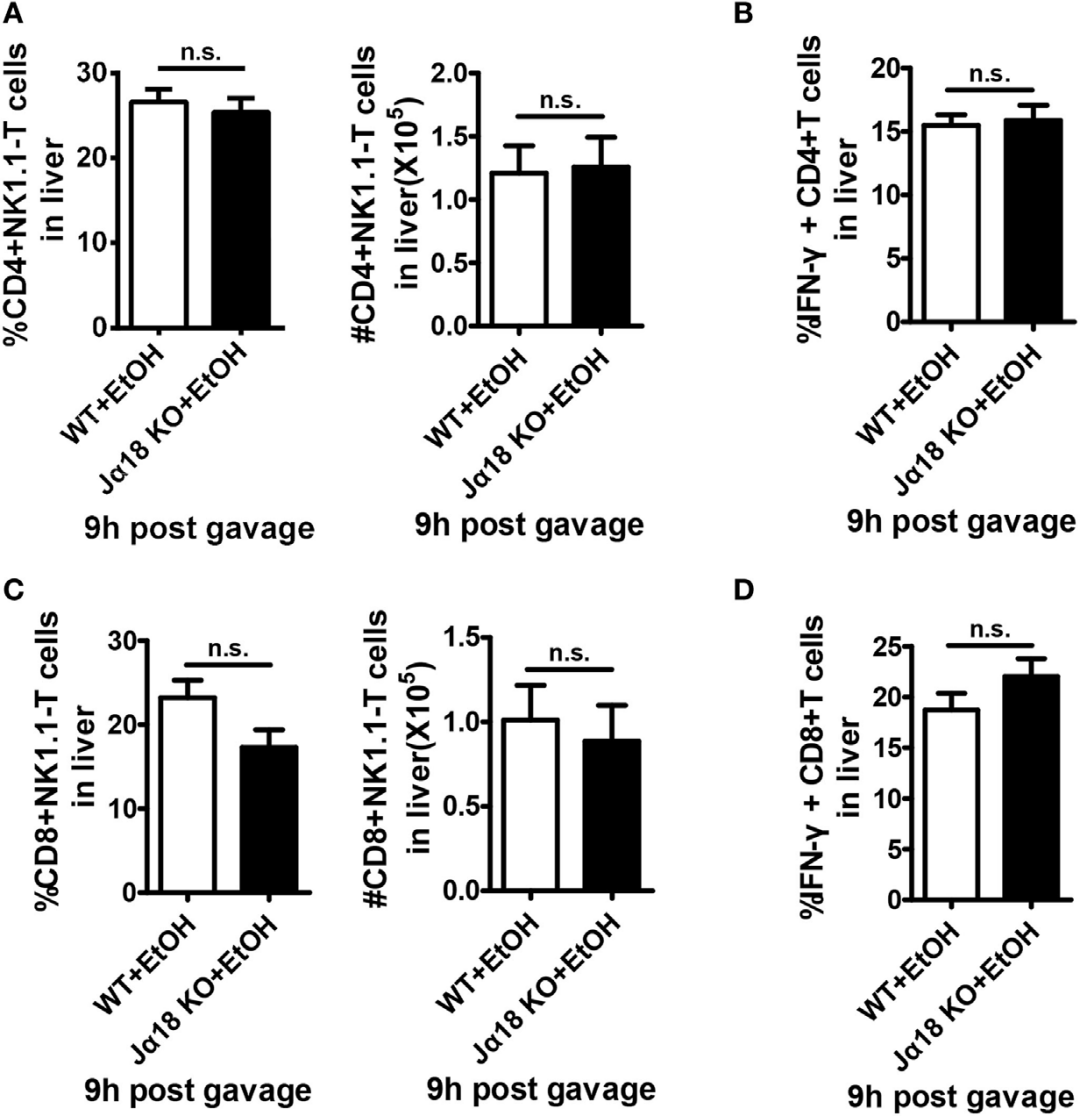
The numbers and interferon-γ (IFN-γ) release of hepatic CD4^+^ and CD8^+^ T cells are not changed in Jα18 KO mice when exposed to ethanol. Wild-type or Jα18 KO mice were fed ethanol diets for 10 days, followed by gavage as described in Figure 1. The mice were sacrificed 9 h after gavage, and liver mononuclear cells were then isolated. The percentages, absolute numbers, and IFN-γ release levels of **(A,B)** CD4^+^ T cells and **(C,D)** CD8^+^ T cells among all leukocytes in the liver were detected by flow cytometry and were statistically analyzed. The data are representative of more than three independent experiments and are shown as the mean ± SEM (*n* = 3–6).

To test this hypothesis, we generated a chronic-binge model in WT and Jα18^−/−^ mice. As predicted, the frequency and absolute number of NK cells in isolated liver MNCs were decreased in ethanol-fed WT mice compared with those in pair-fed controls 9 h after the gavage, whereas the frequency and absolute number of NK cells were significantly higher in ethanol-fed Jα18^−/−^ mice than in WT controls or pair-fed mice (Figures 6A,B). In addition, the degranulation (based on CD107a expression) and IFN-γ release of these NK cells, which were decreased in ethanol-fed WT mice, were almost restored to normal levels in ethanol-fed Jα18^−/−^ mice (Figures 6C,D). Flow cytometric analysis also showed no variances in the frequency, absolute number, and IFN-γ release of hepatic NK1.1^−^ CD4^+^ or NK1.1^−^CD8^+^ T cells in liver MNCs between WT and Jα18^−/−^ mice 9 h after ethanol gavage, excluding the likelihood that the deficiency of iNKT cells induced a systemic increase in lymphocytes in the liver in alcoholic hepatosteatosis, indicating that hepatic iNKT cells are involved in the ethanol-induced inhibition of NK cells, but not conventional CD4^+^/CD8^+^ T cells (Figure 7).

**Figure 8:**
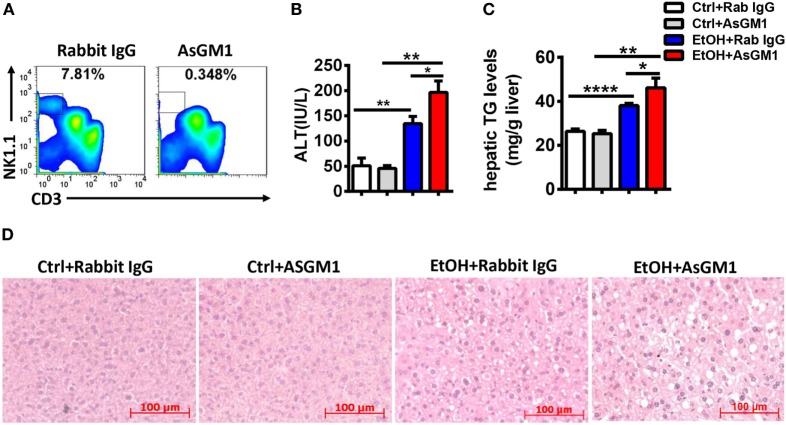
Natural killer (NK) cell deficiency promoted alcoholic hepatosteatosis in Jα18^−/−^ mice. Pair- or ethanol-fed Jα18^−/−^ mice were treated with anti-asialo GM1 or control rabbit IgG once every 3 days as described in Section “Materials and Methods” to deplete NK cells. The mice were euthanized 9 h after gavage. **(A)** Liver mononuclear cells were isolated, and the depletion efficiency was verified by flow cytometry; **(B–D)** liver injury and steatosis were evaluated by measuring **(B)** serum alanine aminotransferase and **(C)** hepatic triglyceride levels and **(D)** liver tissue by hematoxylin and eosin staining (original magnification, 200×). The data are representative of two independent experiments and are shown as the mean ± SEM (*n* = 3–6). *P < 0.05; **P < 0.01; ****P < 0.0001.

To examine whether the increases in the number and functions of NK cells in the liver are required to ameliorate alcoholic hepatosteatosis, Jα18^−/−^ mice undergoing chronic-binge model were treated with an intravenous injection of anti-AsGM1 antibody or rabbit IgG. Liver MNCs were isolated 9 h after gavage and analyzed by flow cytometry to confirm the depletion efficiency of CD3^−^NK1.1^+^ NK cells (Figure 8A). Serum ALT and histologic evaluations revealed that NK cell depletion apparently aggravated alcoholic liver injury and steatosis (Figures 8B–D), suggesting that the increased number and functions of NK cells played crucial roles in protecting Jα18^−/−^ mice from alcoholic hepatosteatosis. In conclusion, these data reveal that iNKT cells play critical roles in inhibiting the accumulation and protective functions of hepatic NK cells and subsequently aggravate the pathogenesis of alcoholic hepatosteatosis.

### IL-10 from iNKT Cells Antagonizes the Protective Role of NK Cells in Alcoholic Hepatosteatosis

**Figure 9:**
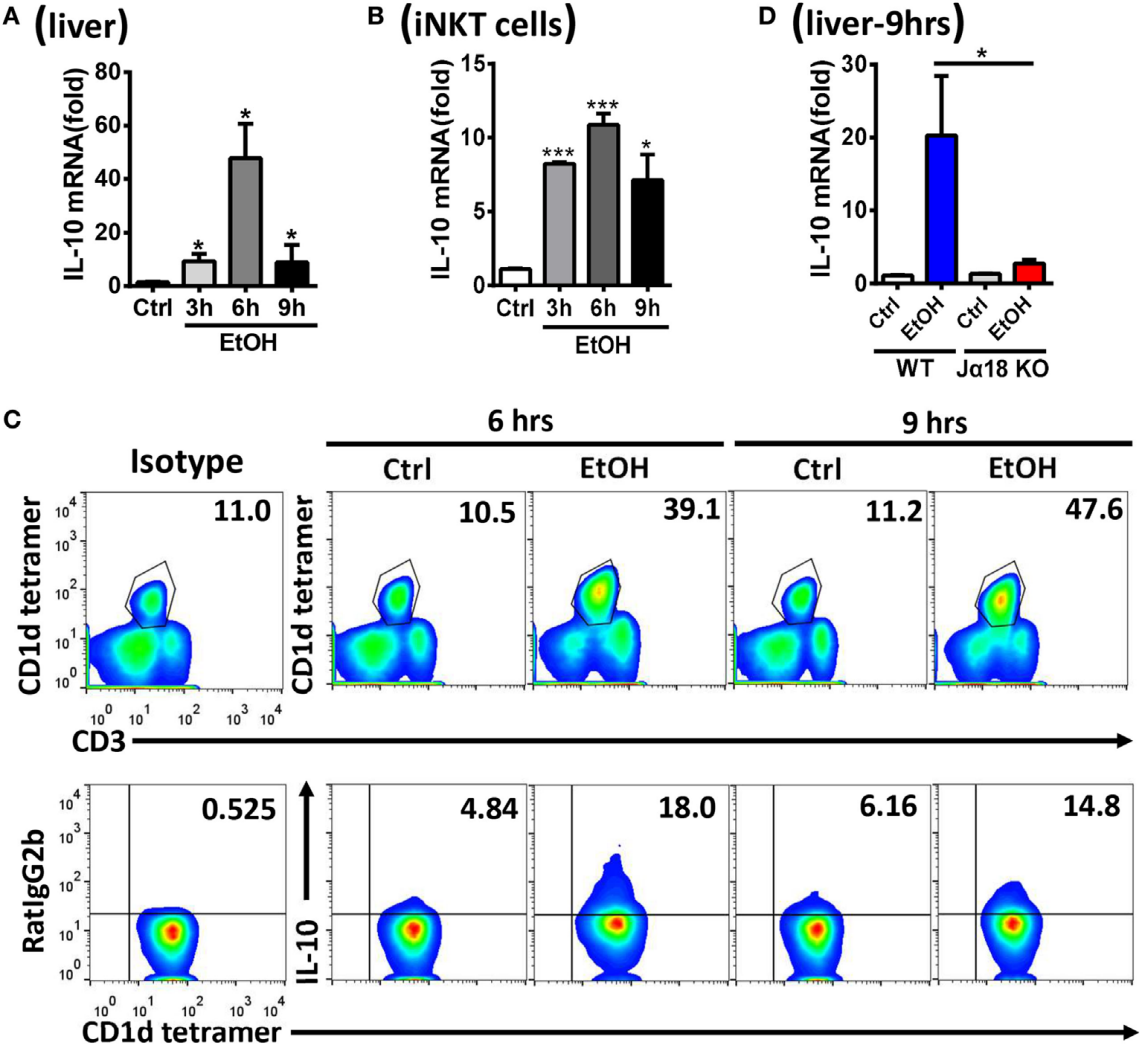
Hepatic invariant natural killer T (iNKT)-derived interleukin-10 (IL-10) was markedly increased after alcohol challenge. **(A–C)** C57BL/6 mice were fed control or ethanol diets for 10 days plus one intragastric dose of EtOH (5 g/kg body weight) or isocaloric dextran-maltose, followed by euthanasia 3, 6, or 9 h after gavage. The mRNA levels of IL-10 in **(A)** liver tissues and **(B)** purified hepatic iNKT cells were evaluated by quantitative PCR (q-PCR). **(C)** Liver mononuclear cells were isolated, and the frequency of CD3^+^CD1d tetramer^+^ iNKT cells among all leukocytes in the liver and the release of IL-10 by iNKT cells were analyzed by flow cytometry. **(D)** Wild-type or Jα18^−/−^ mice were fed control or ethanol diets for 10 days, followed by gavage as described in Figure 3. The mice were euthanized 9 h after gavage, and the mRNA levels of IL-10 in liver tissues were detected by q-PCR. The data are representative of two independent experiments and are shown as the mean ± SEM (*n* = 4). *P < 0.05; ***P < 0.005.

Natural killer T cells are functionally involved in liver disease through the release of multiple cytokines, including both proinflammatory and anti-inflammatory cytokines (11). To investigate how iNKT cells exert inhibitory roles toward NK cells and promote ethanol-induced liver injury and steatosis, the mRNA levels of various potential cytokines, including transforming growth factor-β (TGF-β), IL-4, and IL-10, in liver tissues and sorted hepatic iNKT cells were evaluated at different time points after the gavage of WT mice undergoing chronic-binge model. IL-10, but not TGF-β or IL-4, was obviously upregulated, particularly at 6 h post-ethanol gavage (Figures 9A,B; data not shown). This result was further confirmed through the flow cytometric analysis of IL-10 production in accumulated CD3^+^CD1d tetramer^+^ iNKT cells in liver MNCs without any *in vitro* stimulation at 6 or 9 h after the gavage (Figure 9C). Moreover, the mRNA levels of IL-10 in the liver tissues of Jα18^−/−^ mice receiving chronic-binge ethanol feeding were significantly lower than in the control WT mice, showing expression almost at the normal levels observed in the pair-fed controls (Figure 9D), indicating that iNKT-derived IL-10 was notably increased following excessive alcohol consumption.

**Figure 10:**
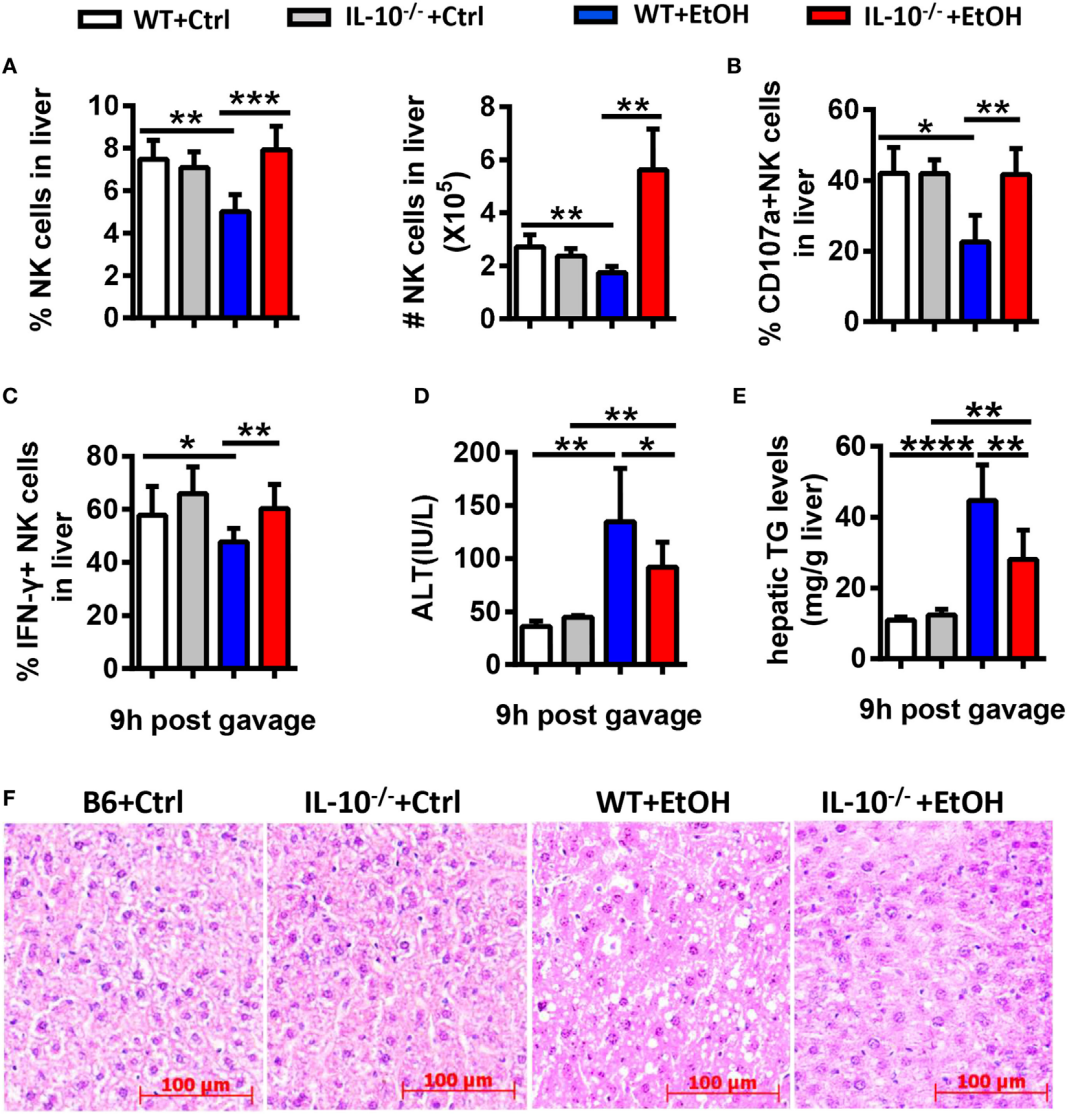
Interleukin-10 (IL-10) deficiency promoted natural killer (NK) cell accumulation and activation and alleviated alcoholic hepatosteatosis. Wild-type or IL-10^−/−^ mice were fed control or ethanol diets for 10 days, followed by gavage, and the mice were euthanized 9 h after gavage. **(A–C)** Liver mononuclear cells were isolated. **(A)** The frequency and numbers of CD3^−^NK1.1^+^ NK cells among all leukocytes in the liver were analyzed by flow cytometry. **(B,C)** Statistical analysis of the percentages of **(B)** CD107a^+^ (with PMA stimulation) and **(C)** interferon-γ^+^ NK cells (with PMA stimulation). **(D–F)** Liver injury and steatosis were evaluated based on **(D)** serum alanine aminotransferase and **(E)** hepatic triglyceride levels and **(F)** liver tissue hematoxylin and eosin staining (original magnification, 200×). The data are representative of two independent experiments and are shown as the mean ± SEM (*n* = 4–10). *P < 0.05; **P < 0.01; ***P < 0.005; ****P < 0.0001.

**Figure 11:**
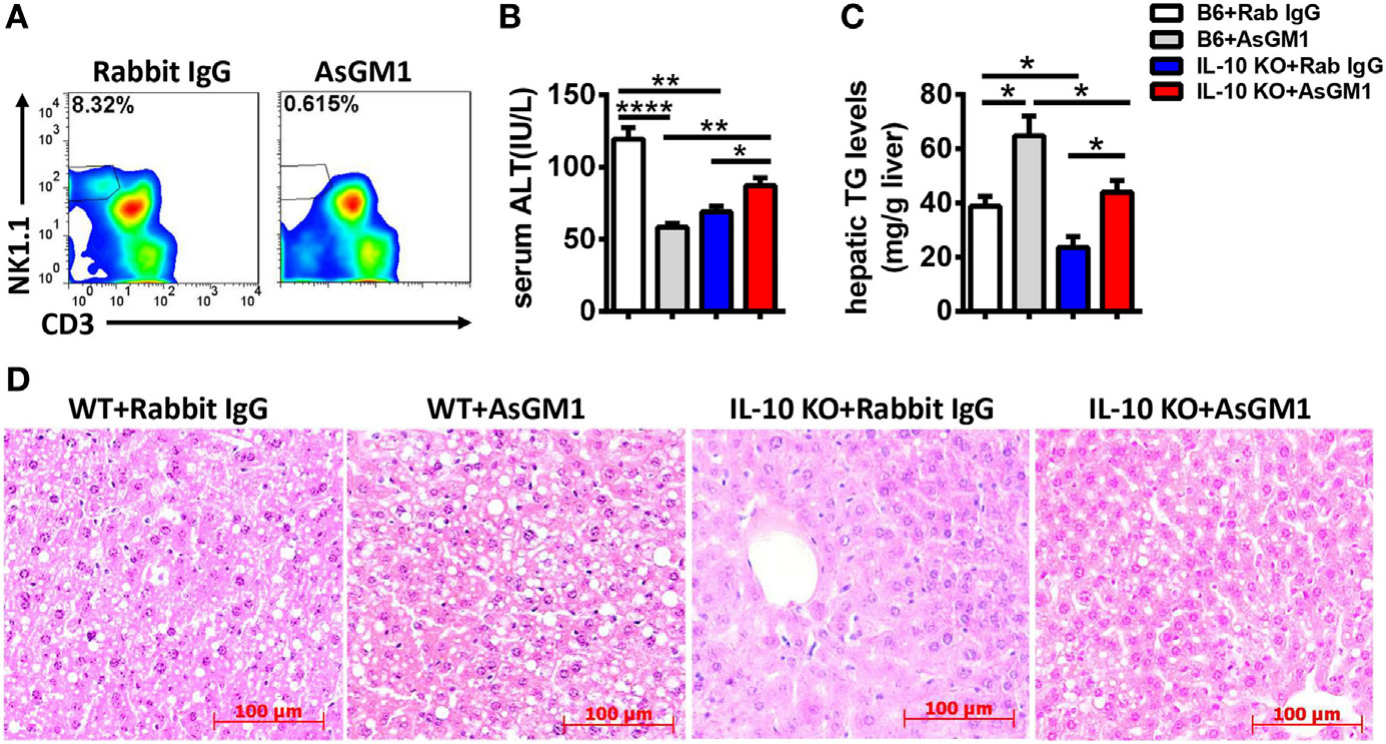
Natural killer (NK) cell deficiency promoted alcoholic hepatosteatosis in interleukin-10 (IL-10) KO mice. Ethanol-fed wild-type or IL-10 KO mice were treated with anti-asialo GM1 or control rabbit IgG once every 3 days as described in Section “Materials and Methods” to deplete NK cells. The mice were euthanized 9 h after gavage. **(A)** Liver mononuclear cells were isolated, and the depletion efficiency was verified by flow cytometry; **(B–D)** liver injury and steatosis were evaluated based on **(B)** serum alanine aminotransferase and **(C)** hepatic triglyceride levels and **(D)** liver tissue hematoxylin and eosin staining (original magnification, 200×). The data are representative of two independent experiments and are shown as the mean ± SEM (*n* = 4–5). *P < 0.05; **P < 0.01; ****P < 0.0001.

Since IL-10 is a well-known inhibitory cytokine that suppresses NK cell recruitment and activation in various diseases (3), we hypothesized that IL-10 plays a key role in the alcohol-induced inhibition of NK cells. To address this hypothesis, we generated a chronic-binge model in WT and IL-10^−/−^ mice. IL-10^−/−^ mice displayed increases in the frequency, absolute number, degranulation, and IFN-γ release of hepatic NK cells compared with control WT mice at 9 h after ethanol gavage (Figures 10A–C). In addition, evaluations of serum ALT levels, hepatic TG, and H&E staining of liver tissue revealed that liver damage and steatosis were obviously alleviated in IL-10^−/−^ mice compared with WT controls (Figures 10D–F). Furthermore, the depletion of NK cells in ethanol-exposed IL-10^−/−^ mice displayed nearly the same result as anti-AsGM1-treated Jα18^−/−^ mice undergoing chronic-binge ethanol consumption (Figure 11). These results demonstrated that IL-10 is required for the suppression of the recruitment and functions of NK cells and promotes alcoholic hepatosteatosis.

**Figure 12:**
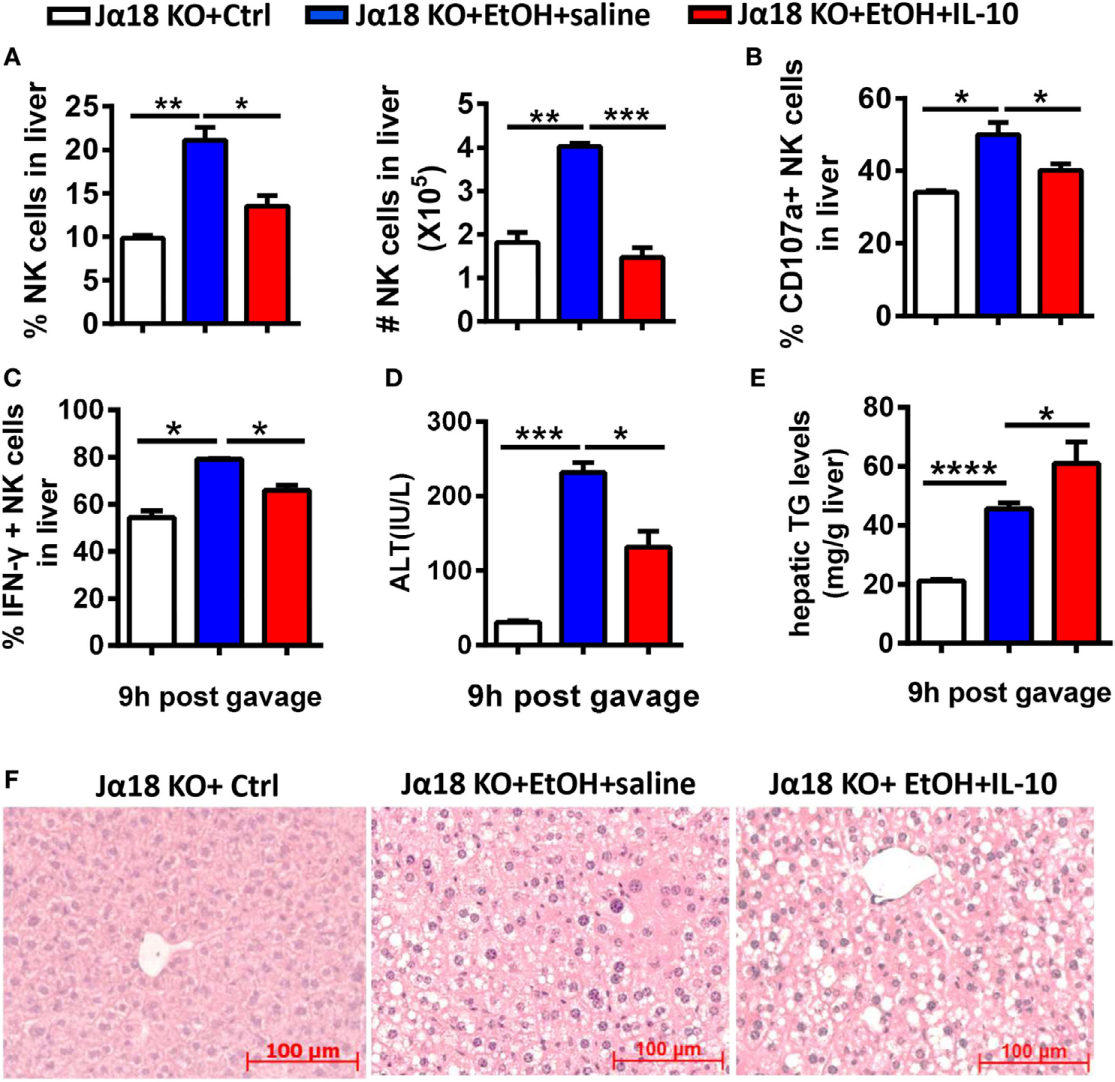
Exogenous interleukin-10 (IL-10) inhibited natural killer (NK) cell activities and promoted liver steatosis in Jα18^−/−^ mice. Jα18^−/−^ mice were treated with daily intraperitoneal injection of 100 ng of recombinant murine IL-10 or an equal volume of PBS along with 10-day ethanol or control diets. The mice were euthanized 9 h after gavage. **(A–C)** Liver mononuclear cells were isolated. **(A)** The frequency and numbers of CD3^−^NK1.1^+^ NK cells among all leukocytes in the liver were analyzed by flow cytometry. **(B,C)** Statistical analysis of the percentages of **(B)** CD107a^+^ (with PMA stimulation) and **(C)** interferon-γ^+^ NK cells (with PMA stimulation). **(D–F)** Liver injury and steatosis were evaluated based on **(D)** serum alanine aminotransferase and **(E)** hepatic triglyceride levels and **(F)** liver tissue hematoxylin and eosin staining (original magnification, 200×). The data are representative of three independent experiments and are shown as the mean ± SEM (*n* = 4–5). *P < 0.05; **P < 0.01; ***P < 0.005; ****P < 0.0001.

To examine whether IL-10 is sufficient for iNKT cells to exert their inhibitory roles, Jα18^−/−^mice undergoing chronic-binge ethanol consumption were treated with a daily intraperitoneal injection of mouse recombinant IL-10 (rmIL-10) or an equal volume of saline. As expected, the frequency, absolute number, degranulation, and IFN-γ release of NK cells in liver MNCs were significantly lower in the rmIL-10-treated mice compared with saline-treated mice at 9 h after ethanol gavage (Figures 12A–C). IL-10 treatment also apparently exacerbated alcoholic liver steatosis, but not liver injury, suggesting that exogenous IL-10 may exert systemic anti-inflammatory roles and suppress the protective function of NK cells against alcohol-induced fatty liver (Figures 12D–F).

**Figure 13:**
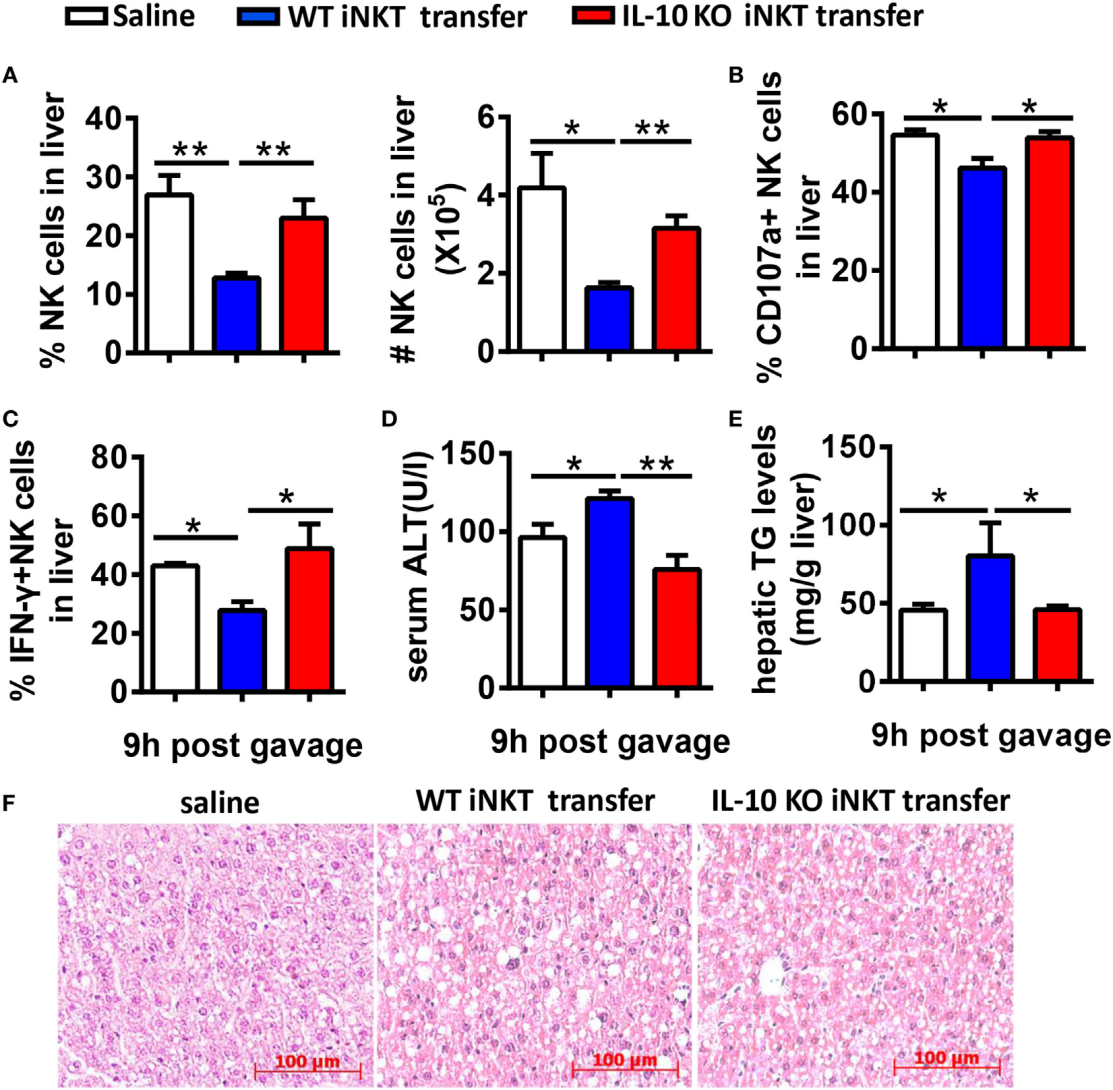
Adoptive transfer of interleukin-10 (IL-10)^−/−^ cells, but not wild-type (WT) invariant natural killer T (iNKT) cells, restored NK cell accumulation, and function in Jα18^−/−^ mice. Jα18^−/−^ mice were fed ethanol diets for 10 days, followed by gavage with a single dose of ethanol and the intrasplenic adoptive transfer of iNKT cells (5 × 10^5^ cells) purified from the livers of WT or IL-10^−/−^ mice. The mice were sacrificed 9 h after gavage. **(A–C)** Liver mononuclear cells were isolated. **(A)** The percentage and absolute number of NK cells among all leukocytes in the liver. **(B)** CD107a expression (with PMA stimulation) and **(C)** interferon-γ release (with PMA stimulation) from hepatic NK cells were detected by flow cytometry and were statistically analyzed. **(D–F)** Liver injury and steatosis were evaluated based on **(D)** serum alanine aminotransferase and **(E)** liver triglyceride levels and **(F)** liver tissue hematoxylin and eosin staining (original magnification, 200×). The data are representative of two independent experiments and are shown as the mean ± SEM (*n* = 3–7). *P < 0.05; **P < 0.01.

To further confirm that IL-10 derived from iNKT cells inhibits the recruitment and activation of NK cells in the liver, we adoptively transferred hepatic iNKT cells from 10-day ethanol-containing diet-fed WT or IL-10^−/−^ mice into Jα18^−/−^ mice undergoing chronic-binge treatment. Flow cytometry analysis confirmed the purity of the isolated iNKT cells (data not shown). As expected, the mice that received WT iNKT cells showed decreases in the frequency, absolute number, degranulation, and IFN-γ release of hepatic NK cells compared with controls (Figures 13A–C), accompanied by significantly more severe alcohol-induced liver injury and steatosis (Figures 13D–F). However, when Jα18^−/−^ mice received the transfer of IL-10-deficient iNKT cells, the number, and functions of NK cells were restored, and the pathogenesis of alcoholic hepatosteatosis was alleviated (Figure 13). Collectively, these results provide evidence that IL-10 produced from a regulatory subset of iNKT cells, called NKT10 cells, as previously reported (15), is responsible for antagonizing the protective roles of hepatic NK cells and therefore contributes to the development of alcoholic liver steatosis.

## Discussion

Relying on their natural cytotoxicity and specific cytokine profiles, NK cells within the liver microenvironment have unique phenotypic features and functional properties (4). In the present study, we provided the first evidence that NK cells protect against alcoholic hepatosteatosis through downregulation of lipid synthesis in hepatocytes through IFN-γ release. These roles of NK cells can be antagonized by a distinct NKT10 cell subset *via* producing IL-10.

Unlike the crosstalk between NK cells and HSCs in alcoholic liver fibrosis, the present study indicated that NK cells may directly affect hepatocytes to exert protective roles against liver steatosis *via* IFN-γ which inhibits lipogenesis-associated gene expression in hepatocytes (Figures 1C,D, 2B,C, 4A and 6C,D), which was further confirmed by using IFN-γ-deficient GKO mice (Figures 3A–C). Notably, GKO mice developed more severe hepatosteatosis and liver injury than NK cell-depleted mice after alcohol consumption, as IFN-γ can also be released by many other immune cell types, not only NK cells (Figures 1B–D and 3A–C). These results reveal another novel critical mechanism by which NK cells prevent liver fibrosis.

Accumulating evidence in recent decades suggests that the interactions between NK cells and other cell types in the liver, such as Kupffer cells, NKT cells, CD4^+^/CD8^+^ T cells, and HSCs, are essentially involved in the changes of the activities of NK cells (7, 25–29). Indeed, though the frequency, number, and functions of NK cells were decreased in WT mice undergoing chronic-binge alcohol consumption, they were significantly increased in ethanol-fed iNKT cell-deficient Jα18^−/−^ mice, indicating that iNKT cells negatively regulate NK cells, leading to alcoholic hepatosteatosis because of losing protection from NK cells (Figures 5 and 6). This result was confirmed by evidence that liver injury and steatosis were aggravated in alcohol consumption-related NK cell-depleted Jα18^−/−^ mice (Figure 8). The roles of iNKT-IL10-NK-IFN-γ axis in alcoholic hepatosteatosis are meaningful and deserve further study in other liver diseases.

Natural killer cell activation or inhibition is driven through certain cytokines. For example, IFN-α/β is considered the most potent activator of NK cell cytotoxicity, IL-12 induces IFN-γ production in NK cells associated with IL-18, and IL-15 promotes NK cell proliferation (3, 30, 31). Analogous to NK cells, activated NKT cells can crack target cells and damage the body tissues through cell cytotoxicity and can regulate various immune responses that rely on the strong secretion of multiple cytokines, including Th1, Th2, and Th17 cytokines (32). Using a chronic-binge model, we showed that IL-10, an iNKT-derived inhibitory cytokine, may be responsible for the alcohol-induced suppression of NK cell functions in the liver, since IL-10 expression was obviously upregulated in ethanol-exposed hepatic iNKT cells (Figure 9), and the reduced number and functions of hepatic NK cells were restored to normal levels in IL-10^−/−^ mice undergoing chronic-binge alcohol consumption, and liver steatosis and injury were attenuated compared with WT controls (Figure 10). Interestingly, the treatment with exogenous IL-10 obviously inhibited the increase of hepatic NK cell number and function in ethanol-fed Jα18^−/−^ mice, and promoted alcohol-induced hepatosteatosis (Figures 12A–C,E,F). Importantly, ethanol-fed Jα18^−/−^ mice receiving the adoptive transfer of hepatic iNKT cells purified from WT but not IL-10^−/−^ mice, showed reduced numbers and functions of hepatic NK cells and developed more severe alcohol-induced hepatosteatosis, confirming that iNKT cells exert regulatory roles on NK cells through IL-10 (Figure 13). However, how IL-10 acts on NK cells still remains elusive.

In conclusion, this study provided the first demonstration that NK cells can protect against alcohol-induced fatty liver through IFN, which directly downregulates lipogenesis in hepatocytes, and alcohol consumption also induces a regulatory NKT10 subset of hepatic iNKT cells to release high levels of IL-10 which subsequently inhibits recruitment and functions of NK cells, to antagonize the protective effects of NK cells. The result obtained reveals a new mechanism of NK-NKT cell interplay through which the pathogenesis of alcoholic hepatosteatosis is controlled or out of order.

## Ethics Statement

All animal welfare and experimental procedures were in accordance with National Institutes of Health Guide for the Care and Use of Laboratory Animals, and the protocols used were approved by the Animal Ethics Committee of the University of Science and Technology of China, Hefei, China.

## Author Contributions

KC performed the experiments and acquisition and analysis of data, and drafted the manuscript. GY did parts of the experiments and data acquisition. XZ provided technical assistants. LB provided some gene knockout mice and fruitful discussion. HW participated in designing experiments. ZT and RS started the study and participated in the experimental design, data analysis, and the paper writing.

## Conflict of Interest Statement

The authors declare that the research was conducted in the absence of any commercial or financial relationships that could be construed as a potential conflict of interest.

## Abbreviations

ALD, alcoholic liver disease; NK, natural killer; IFN, interferon; AsGM1, asialo GM1; IL, interleukin; TNF-α, tumor necrosis factor-α; HSCs, hepatic stellate cells; TCR, T cell receptor; iNKT, invariant natural killer T; MNCs, mononuclear cells; EAE, experimental autoimmune encephalomyelitis; ALT, alanine aminotransferase; TG, triglyceride; TGF-β, transforming growth factor-β.
